# Association between functional lactase variants and a high abundance of *Bifidobacterium* in the gut of healthy Japanese people

**DOI:** 10.1371/journal.pone.0206189

**Published:** 2018-10-19

**Authors:** Kumiko Kato, Sachiko Ishida, Masami Tanaka, Eri Mitsuyama, Jin-zhong Xiao, Toshitaka Odamaki

**Affiliations:** 1 Next Generation Science Institute, Morinaga Milk Industry Co., LTD., Kanagawa, Japan; 2 Business Planning Group, Next Generation Science Institute, DeNA Life Science, Inc., Tokyo, Japan; 3 R&D Group, DeNA Life Science, Inc., Tokyo, Japan; University of Illinois at Urbana-Champaign, UNITED STATES

## Abstract

Previous studies have shown that Japanese people exhibit a higher abundance of *Bifidobacterium* compared to people from other countries. Among the possible factors affecting the gut microbiota composition, an association of functional lactase gene variants with a higher abundance of *Bifidobacterium* in the gut has been proposed in some reports. However, no Japanese subjects were included in these studies. In this study, we investigated the possible contribution of functional lactase loci to the high abundance of *Bifidobacterium* in Japanese populations. Based on a data analysis assessing 1,068 healthy Japanese adults, a number of subjects is at least seven times greater than that reported in available online data. all subjects possessed CC genotype at rs4988235 and the GG at rs182549, which are associated with low lactase activity. We observed a positive correlation between dairy product intake and *Bifidobacterium* abundance in the gut. Considering previous reports, which revealed that four additional functional lactase loci, rs145946881, rs41380347, rs41525747 and rs869051967 (ss820486563), are also associated with low lactase activity in Japanese people, our findings imply the possible contribution of host genetic variation-associated low lactase activity to the high abundance of *Bifidobacterium* in the Japanese population.

## Introduction

The life expectancy of Japanese population is one of the longest life expectancies of any country in the world[1]. The underlying causes of long life in Japan have been debated, and the possibilities include good hygiene, a high level of health consciousness in Japan, and the Japanese diet[2]. In previous reports, Japanese healthy adults exhibited a higher abundance of *Bifidobacterium* compared to people from other countries[3–5]. Considering that *Bifidobacterium* naturally inhabits the human gastrointestinal tract (GIT) and is thought to play pivotal roles in maintaining human health[6], the higher abundance of this genus seems to be another potential reason for long life in Japan. The proportion of *Bifidobacterium* in the gut microbiota is affected by many factors, such as host age[4], stress[7] and diet[8].

Host genetic variations related to fucosyltransferase 2[9] and lactase[10–13] have been reported to contribute to *Bifidobacterium* abundance. In particular, the functional lactase gene (*LCT*) variant rs4988235 has been associated with *Bifidobacterium* abundance in the gut in multiple reports[14–16]. The presence of the CC genotype at rs4988235 is related to low lactase activity and milk indigestibility in adulthood. Because *Bifidobacterium* assimilates lactose as a preferred carbon source for growth, it is reasonable that subjects with the CC genotype at this locus have a higher *Bifidobacterium* abundance in their gut. The locus rs4988235 is mainly reported as a characteristic locus related to lactase activity in Europeans. However, this single locus is insufficient to explain the frequency of the lactase phenotype present in various populations worldwide[11]. Another variant, the GG genotype at rs182549, has been reported to contribute to lactase persistence in Japanese-Brazilian and Chinese populations, whose genetic backgrounds are related more closely to those of the Japanese population than to those of the European population[11]. Therefore, the rs182549 locus might also be an important contributor to the lactase activity in the Japanese population.

Here, we investigated the associations between these two single nucleotide polymorphisms (SNPs), which have been reported as functional lactase variants, and the proportion of *Bifidobacterium* in the gut microbiota of 1,068 healthy Japanese adults. Furthermore, information for an additional four functional lactase variants that were reported previously[12,13] but were missing from our data set was also included using Japanese genome data obtained from two previous studies[13,17].

## Results/Discussion

Of 1,250 participants enrolled in this study, a total of 182 participants were removed from data analysis due to refusal to participate (n = 64), pregnancy or lactation (n = 7), medication use within the last two weeks (n = 96), and mismatch with the criteria for quality control of genotypic data (n = 15, see materials and methods). Finally, a total of 1,068 healthy Japanese subjects were selected (S1 Fig).

The abundance of *Bifidobacterium* in the Japanese gut microbiota was confirmed by 16S ribosomal RNA gene sequencing. A total of 9,285,977 high-quality paired sequences were obtained from 1,068 samples, and 8,695±2,255 (average±standard deviation) reads per sample were generated. The average of *Bifidobacterium* abundance was 6.3±7.8%, which was lower than that in previous Japanese reports (17.9±15.2%[3], 10.5±11.0%[4], 13.6±17.7%[5]). This discrepancy might be due to differences in faecal samplings, DNA extraction and analysis methods including the primers used[5,18,19], in addition to the distribution of the population in each study. Nevertheless, the percentage of *Bifidobacterium* abundance in this study is higher than those for data on subjects in many other countries[3,20–22]. A previous report showed that *Bifidobacterium* abundance was less than 5% in 9 of 11 countries, with an average of 3.89±5.45%[3]. The relatively higher abundance of *Bifidobacterium* in the Japanese gut microbiota was confirmed in our data.

We then investigated the genotype frequencies of two functional *LCT* variants, rs4988235 and rs182549, for lactase persistence. The genotyping results showed that these two variants were monomorphic: all subjects had a CC genotype at rs4988235 and GG genotype at rs182549 (Table 1), which are associated with low lactase activity. These genotypic frequencies for the two variants were confirmed in another 42 and 104 Japanese samples from the previous report [13] and the 1000 Genomes Project [17], respectively. In addition to the two variants, four other *LCT* variants, rs145946881, rs41380347, rs41525747 and rs869051967 (ss820486563), have also been reported to affect lactase activity[12,13]. Our genotype dataset unfortunately lacked information concerning these four variants; however, we observed no variation at these SNPs in the Japanese population data from two previous studies[13,17](Table 1). Table 2 shows the frequencies of LCT non-persistent genotypes at these six SNPs in different populations. Only the East Asian population, including Japanese population, has no variation at all of these SNPs. Based on these findings, all available data related to functional lactase variants regarding the Japanese genome indicated that low lactase activity in the Japanese population seems to contribute to the higher abundance of *Bifidobacterium* in the Japanese population.

**Table 1:** Frequencies of LCT non-persistent genotypes in the Japanese population.

rsID	rs4988235	rs182549	rs145946881	rs41380347	rs41525747	rs869051967
						(ss820486563)
Chr.	2	2	2	2	2	2
position	136608646	136616754	136608746	136608651	136608643	136608745
	LCT-13910 C>T	LCT-22018 G>A	LCT-14010 G>C	LCT-13915 T>G	LCT-13907 C>G	LCT-14009 T>G
Genotype of LCT non-persistence	CC	GG	GG	TT	CC	TT
This study	100%	100%	N/A*^3^	N/A	N/A	N/A
(n = 1068)	(1068/1068) *^1^	(1061/1061)*^2^				
Reference [12]	100%	N/A	100%	100%	100%	100%
(n = 42)	(42/42)		(42/42)	(42/42)	(42/42)	(42/42)
Reference [17]	100%	100%	100%	100%	N/A	N/A
(n = 104)	(104/104)	(104/104)	(104/104)	(104/104)		

*1 Non-persistence/persistence subject number

*2 No rs182549 data in seven subjects

*3 N/A, not available

**Table 2:** Frequencies of LCT non-persistent genotypes in the different population based on reference [17].

rsID	rs4988235	rs182549	rs145946881	rs41380347	rs41525747	rs869051967
						(ss820486563)
Chr.	2	2	2	2	2	2
position	136608646	136616754	136608746	136608651	136608643	136608745
	LCT-13910 C>T	LCT-22018 G>A	LCT-14010 G>C	LCT-13915 T>G	LCT-13907 C>G	LCT-14009 T>G
Genotype of LCT non-persistence	CC	GG	GG	TT	CC	TT
All	75.60%	75.40%	99.40%	99.90%	N/A*^2^	N/A
(n = 2504)	(1893/2504)*^1^	(1887/2504)	(2490/2504)	(2501/2504)		
African	95.20%	95.20%	97.90%	100%	N/A	N/A
(n = 661)	(629/661)	(629/661)	(647/661)	(661/661)		
American	63.10%	62.80%	100%	99.10%	N/A	N/A
(n = 347)	(219/347)	(218/347)	(347/347)	(344/347)		
East Asian	100%	100%	100%	100%	N/A	N/A
(n = 504)	(504/504)	(504/504)	(504/504)	(504/504)		
European	30.60%	30.40%	100%	100%	N/A	N/A
(n = 503)	(154/503)	(153/503)	(503/503)	(503/503)		
South Asian	79.10%	78.30%	100%	100%	N/A	N/A
(n = 489)	(387/489)	(383/489)	(489/489)	(489/489)		

*1 Non-persistence/persistence subject number

*2 N/A, not available

**Fig 1:**
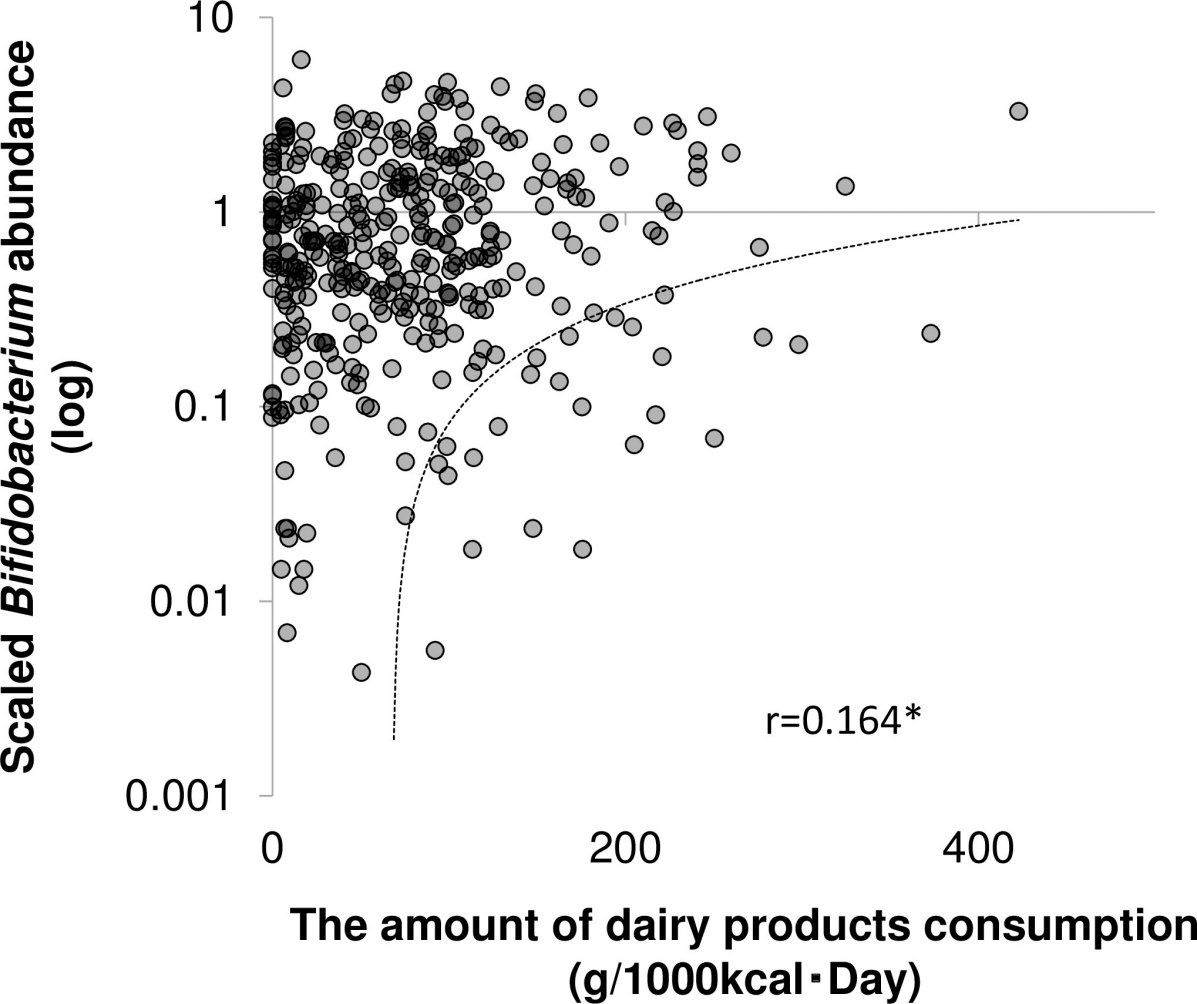
Relationship between dairy product intake and *Bifidobacterium* abundance in the gut. *p<0.01, Spearman’s correlation test. Dot line represents a linearized approximation to the data.

A previous study showed a positive correlation between *Bifidobacterium* abundance in the European population and the amount of dairy product consumption only in subjects with the GG genotypes at rs4988235 on the reverse strand, which is equivalent to the CC genotype on the forward strand [15]. This report indicates that the relatively higher amount of lactose contained in dairy products can directly reach the gut and be available to members of the genus *Bifidobacterium*, due to the low lactase activity. We therefore investigated the association between dairy product intake and *Bifidobacterium* abundance using 1,068 Japanese samples, all of which homogeneously presented the CC genotype. A brief-type diet history questionnaire (BDHQ) showed that the average dairy product consumption in the enrolled subjects was 67.9±61.4 g/1,000 kcal. A significant positive correlation was observed between the amount of dairy consumed and the relative abundance of *Bifidobacterium* in the gut (r = 0.164, p<0.01, Fig 1 and S1 Table), suggesting an association between the higher amount of lactose derived from dairy products and the higher abundance of *Bifidobacterium* in the gut. Considering that the influence of diet on the gut microbiota is very complex and the fact that lactose does not represent a selective growth substrate for bifidobacteria, the monophyletic lactase functional variants in Japanese people may partially contribute to the significant positive correlation between the amount of diet consumption of lactose and the abundance of *Bifidobacterium* in the gut. Nevertheless, our large scale survey of Japanese confirmed the observation, that subjects in the European population with the GG genotypes at rs4988235 could have increased *Bifidobacterium* abundance in the gut by consuming lactose [15].

In conclusion, we demonstrated the association of six lactase functional variants with the relative abundance of *Bifidobacterium* in the Japanese gut using our large data set of 1,068 subjects as well as 146 Japanese genomes derived from two previous reports[13,17]. Our large scale data revealed that there was no variation in the SNPs associated with low lactase activity in the Japanese populations. Referring to a previous report, approximately 85% of Japanese people are reportedly lactose intolerant[23]. The underlying reason for the 15% population gap observed between the phenotype and genotype for lactase activity is unclear. A previous report has suggested that three *LCT* variants, rs41525747, rs41380347 and rs145946881, which differ from the variants that contributed to lactase activity in European and American populations, are effective functional variants in Africa and the Middle East[11]. Since the effective locus is thought to vary between population origins, there might be undiscovered lactase functional variants in Japanese populations.

**Fig 2:**
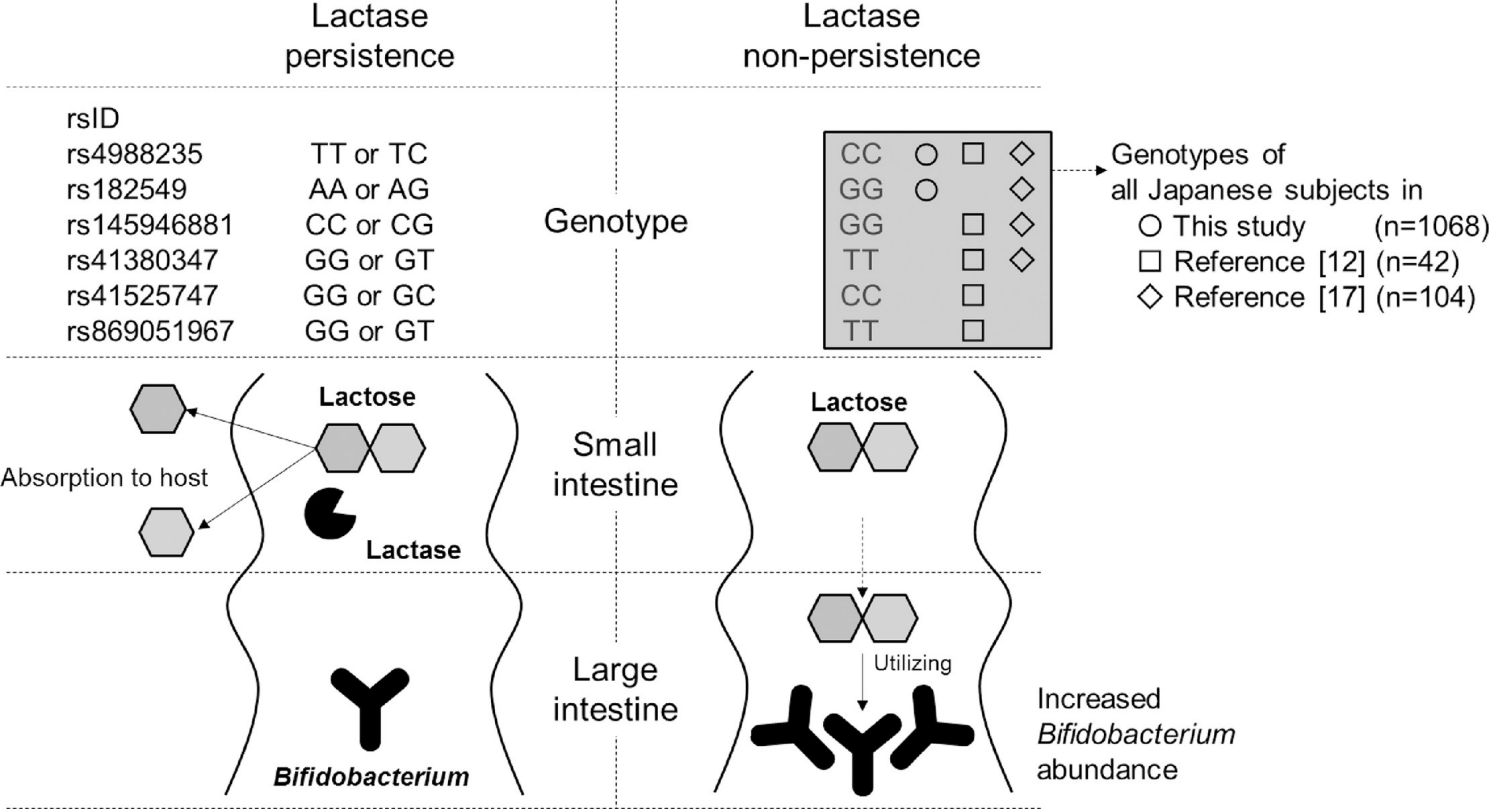
Summary of *LCT* functional variants. The large data set suggest a possible contribution of monophyletic lactase variants to the higher abundance of *Bifidobacterium* in the Japanese population.

Our results suggest a possible contribution of monophyletic lactase variants to the higher abundance of *Bifidobacterium* in the Japanese population (Fig 2). However, since lactose is not a selective growth substrate for *Bifidobacterium* in the gut, we should consider other possible contributing factors, such as the various types of soluble dietary fibres in Japanese diet. Further studies are needed to understand the mechanism by which the gut microbiota composition is established.

## Material and method

### Study subjects and sample collection

A total of 1,250 healthy Japanese adults were enrolled in this study as part of MYCODE Research, a research platform based on customers of MYCODE (DeNA Life Science Inc., Tokyo, Japan), a personal genome service in Japan. A total of 182 subjects were removed from the data analysis based on the following criteria (see also S1 Fig): 64 subjects declined participation, seven subjects were pregnant or lactating women, 96 subjects had taken medication within the last two weeks, and 15 subjects did not meet the criteria for quality control of the genotypic data as described in detail below. Finally, the 1,068 participants consisted of 541 women and 527 men with a median age of 41 years (range 20–64 years) and median body mass index of 21.4 kg/m^2^ (inter-quartile range 19.7–23.7 kg/m^2^).

The entire study was approved by both the ethics committee of DeNA Life Science Inc. (protocol #20160727_1) and the Institute of Medical Science, University of Tokyo (protocol #28-29-1125) (Tokyo, Japan).

Written informed consent was initially obtained for MYCODE Research covering diverse genomic research comprehensively. Then, additional informed consent for this specific study was obtained from all subjects on the MYCODE website.

Saliva samples were collected for MYCODE genetic testing, and these genetic data were used for this study. All participants submitted their own stool samples for gut microbiota analyses. A stool sample aliquot was mixed with 1 ml of guanidine thiocyanate (GuSCN) solution (TechnoSuruga Laboratory Co., Ltd, Shizuoka, Japan)[24] and was transported to the laboratory by postal mail at room temperature. Immediately upon receipt, the faecal samples were stored at −80°C until the day of analysis.

### Genotyping and quality control

Genotyping of SNPs, was performed using either an Infinium OmniExpress-24+ BeadChip or a Human OmniExpress-24+ BeadChip (Illumina Inc., San Diego, CA, United States). Based on the genotypic data, a total of 15 subjects were removed in the process of quality controls using PLINK version 1.9[25] as follows: two subjects with call rates under 95% which indicated low reliability of their genotyping results; nine subjects who were one of a pair with a proportion of identical by descent (IBD) >0.185; which indicates kinship; one subject showing a discordance between self-reported sex and genotyped sex; and three subjects determined to have non-Japanese ancestry by principal component analysis.

### DNA extraction from faecal samples

DNA extraction from human faecal samples was performed using the bead-beating method as previously described[4] with some modifications. Briefly, 500 μl of faecal sample in GuSCN solution was vigorously vortexed with glass beads (300 mg; 0.1 mm in diameter) and 500 μl of buffer-saturated phenol using a Multi-Beads Shocker (Yasui Kikai Co., Osaka, Japan) at a speed of 2,700 rpm for 180 s. After centrifugation at 10,000×g for 10 min, 400 μl of the supernatant was extracted with phenol-chloroform, and 250 μl of the supernatant was precipitated with isopropanol. The purified DNA was suspended in 1,000 μl of Tris-EDTA buffer (pH 8.0).

### Sequencing and data processing of bacterial 16S rRNA sequences

16S rRNA gene sequencing was performed as previously described with minor modifications[4]. Briefly, the V3-V4 region of the bacterial 16S rRNA gene was amplified by PCR in triplicate using the TaKaRa Ex Taq HS Kit (TaKaRa Bio, Shiga, Japan) and the primer sets Tru357F (5′-CGCTCTTCCGATCTCTGTACGGRAGGCAGCA G-3′) and Tru806R (5′-CGCTCTTCCGATCTGACG- GACTACHVGGGTWTCTAAT-3′) with the following program: preheating at 94°C for 3 min; 30 cycles of denaturation at 94°C for 30 s, annealing at 50°C for 30 s and extension at 72°C for 30 s; and terminal extension at 72°C for 5 min. A 1-μl sample of the combined PCR products was amplified with barcoded primers adapted for Illumina MiSeq sequencing: Fwd 5′-AATGATACGGCGACCACCGAGATCTACACXXXXXXXXACACTCTTTCCCTACACGACGCTCTTCCGATCTCTG-3’ and Rev 5′-CAAGCAGAAGACGGCATACGAGATXXXXXXXXGTGACTGGAGTTCAGACGTGTGCTCTTCCGATCTGAC-3′, where X represents a barcode base. Amplification was performed according to the program described above except only eight cycles were performed. The products were purified and quantified by a QIAquick PCR Purification Kit (Qiagen, Valencia, CA, United States) and Quant-iT PicoGreen dsDNA Assay Kit (Thermo Fisher Scientific, Waltham, MA, United States) according to the manufacturer’s protocols. Equal amounts of amplicons were pooled and purified with the GeneRead Size Selection Kit (Qiagen) according to the manufacturer’s protocol. The pooled libraries were sequenced with an Illumina MiSeq instrument and the MiSeq v3 Reagent Kit (Illumina Inc., San Diego, CA, United States).

After acquiring the Illumina paired-end reads, the Bowtie-2 program[26] (ver. 2–2.2.4) was used to remove reads mapped to the PhiX 174 sequence and the Genome Reference Consortium human build 38 (GRCh38). Thereafter, the 3’ region of each read with a PHRED quality score of less than 17 was trimmed. Trimmed reads less than 150 bp in length with an average quality score of less than 25 or those lacking paired reads were also removed. The trimmed paired-end reads were combined by the fastq-join script in EA-Utils[27] (ver. 1.1.2–537). Potential chimeric sequences were removed by reference-based chimaera checking in USEARCH[28] (ver. 5.2.32) and the Genomes OnLine Database (GOLD) (http://drive5.com/otupipe/gold.tz).

Non-chimeric sequences were analysed via the QIIME software package version 1.8.0[29,30]. For genus-level analysis, the sequences were assigned to operational taxonomic units (OTUs) by open-reference OTU picking[31] with a 97% pairwise identity threshold and the Greengenes reference database[32].

### The frequencies of *LCT* variants in different populations around the world

The frequencies of *LCT* variants in Japanese, African, American, East Asian, European, and South Asian populations were obtained from the Phase 3 1000 Genomes Project data (http://phase3browser.1000genomes.org/index.html)[17].

### Assessment of dairy product intake with a BDHQ

A BDHQ[33] was used to assess food intake habits during the month before sample collection.

To estimate the amount of lactose contained in an individual’s diet, we used combined data with the categories “normal-fat milk” and “low-fat milk” (designated here as dairy products), after energy adjustment using the density method.

### Statistical analysis

All analyses were performed using the IBM SPSS Statistics, version 22.0, statistical software package (IBM Corp., Armonk, NY, USA). Intergroup differences in the composition of *Bifidobacterium* were analysed using the Kruskal-Wallis test. Spearman’s correlation coefficient was used to determine the relationship of *Bifidobacterium* abundance with the amount of dairy product intake. For all tests, p<0.05 was considered statistically significant.

## Supporting information

S1 Fig Flow chart of this study. (TIF)

S1 Table Amount of dairy product consumption and *Bifidobacterium* abundance in the gut. (DOCX)
