# Supplementary figures and images for: Association between functional lactase variants and a high abundance of Bifidobacterium in the gut of healthy Japanese people
Source: PLoS One. 2018 Oct 19;13(10):e0206189. doi: 10.1371/journal.pone.0206189 (PMC6195297; doi:10.1371/journal.pone.0206189)

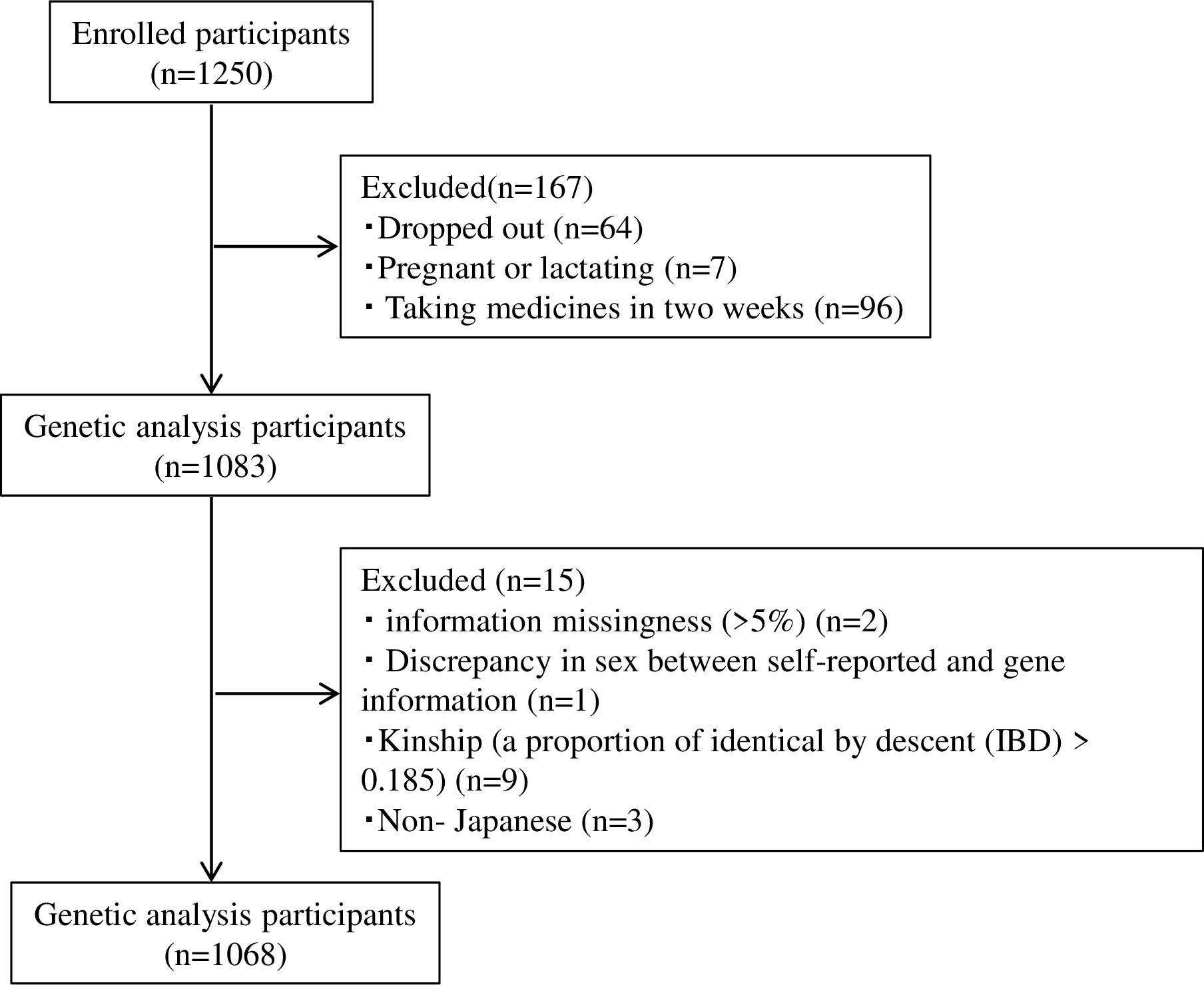

Supplement: S1 Fig — (TIF) [file pone.0206189.s001.tif]
