# Supplementary material for: Association between functional lactase variants and a high abundance of Bifidobacterium in the gut of healthy Japanese people
Source: PLoS One. 2018 Oct 19;13(10):e0206189. doi: 10.1371/journal.pone.0206189 (PMC6195297; doi:10.1371/journal.pone.0206189)
Supplement: S1 Table — (DOCX) [file pone.0206189.s002.docx]

**S1 Table. Dairy product consumption and the *Bifidobacterium* abundance of each subject.**

| SampleID | Dairy product consumption (g/1000kcal) | Bifidobacterium abundance (%) |
| --- | --- | --- |
| 0001 | 0 | 4.63 |
| 0002 | 69.25 | 0.63 |
| 0003 | 4.76 | 0.81 |
| 0004 | 126.52 | 8.21 |
| 0005 | 92.9 | 4.29 |
| 0006 | 111.17 | 0.89 |
| 0007 | 100.36 | 20.27 |
| 0008 | 220.81 | 8.18 |
| 0009 | 6.53 | 13.5 |
| 0010 | 33.77 | 0.89 |
| 0011 | 262.92 | 1.34 |
| 0012 | 96.22 | 4.62 |
| 0013 | 172.21 | 16.1 |
| 0014 | 123.55 | 11.89 |
| 0015 | 119.5 | 2.51 |
| 0016 | 0 | 15.19 |
| 0017 | 128.6 | 4.73 |
| 0018 | 229.31 | 0.06 |
| 0019 | 42.6 | 13.38 |
| 0020 | 92.61 | 12.32 |
| 0021 | 278.46 | 1.81 |
| 0022 | 8.73 | 0.05 |
| 0023 | 71.92 | 17.74 |
| 0024 | 10.68 | 2.54 |
| 0025 | 13.7 | 2.07 |
| 0026 | 85.52 | 1.57 |
| 0027 | 7.19 | 20.82 |
| 0028 | 11.07 | 13.89 |
| 0029 | 21.11 | 1.58 |
| 0030 | 75.39 | 7.18 |
| 0031 | 100.2 | 2.74 |
| 0032 | 0 | 11.01 |
| 0033 | 73.34 | 2.21 |
| 0034 | 43.84 | 0.38 |
| 0035 | 38.2 | 12.23 |
| 0036 | 40.19 | 22.64 |
| 0037 | 7.42 | 2.36 |
| 0038 | 53.73 | 21.68 |
| 0039 | 17.06 | 5.39 |
| 0040 | 0 | 0.39 |
| 0041 | 6.83 | 27.98 |
| 0042 | 100.39 | 9.61 |
| 0043 | 81.3 | 5.75 |
| 0044 | 77.32 | 18.57 |
| 0045 | 45.43 | 8.4 |
| 0046 | 163.74 | 0.71 |
| 0047 | 4.68 | 7.48 |
| 0048 | 111.38 | 11.14 |
| 0049 | 0 | 7.68 |
| 0050 | 58.1 | 2.24 |
| 0051 | 12.69 | 9.08 |
| 0052 | 37.35 | 3.55 |
| 0053 | 12.19 | 10.15 |
| 0054 | 9.16 | 2.19 |
| 0055 | 240.9 | 22.82 |
| 0056 | 63.61 | 6.03 |
| 0057 | 35.42 | 0.08 |
| 0058 | 194.1 | 4.6 |
| 0059 | 95.87 | 6.62 |
| 0060 | 24.61 | 12.26 |
| 0061 | 49.38 | 7.93 |
| 0062 | 131.87 | 2.07 |
| 0063 | 73.97 | 43.24 |
| 0064 | 57.31 | 2.38 |
| 0065 | 60.93 | 0.6 |
| 0066 | 19.46 | 6.55 |
| 0067 | 0 | 1.63 |
| 0068 | 278.1 | 8.54 |
| 0069 | 0 | 2.43 |
| 0070 | 31.22 | 2.52 |
| 0071 | 210.59 | 5.08 |
| 0072 | 77.54 | 9.78 |
| 0073 | 103.35 | 15.45 |
| 0074 | 88.31 | 22.69 |
| 0075 | 89.36 | 0.85 |
| 0076 | 123.39 | 12.77 |
| 0077 | 63.51 | 5.85 |
| 0078 | 96.38 | 0.87 |
| 0079 | 23.25 | 11.63 |
| 0080 | 11.75 | 1.16 |
| 0081 | 52.06 | 11.16 |
| 0082 | 13.61 | 6.18 |
| 0083 | 102.68 | 0.29 |
| 0084 | 81.56 | 3.2 |
| 0085 | 137.42 | 3.12 |
| 0086 | 12.77 | 4.65 |
| 0087 | 8.59 | 2.48 |
| 0088 | 11.19 | 0.04 |
| 0089 | 4.11 | 10.86 |
| 0090 | 112.95 | 0.02 |
| 0091 | 72.17 | 12.68 |
| 0092 | 0 | 1.15 |
| 0093 | 114.49 | 4.26 |
| 0094 | 133.93 | 2.31 |
| 0095 | 0 | 0.53 |
| 0096 | 60.29 | 23.71 |
| 0097 | 149.82 | 8.16 |
| 0098 | 111.73 | 0.17 |
| 0099 | 74.87 | 0.4 |
| 0100 | 39.76 | 10.73 |
| 0101 | 16.33 | 53.82 |
| 0102 | 97.22 | 0.54 |
| 0103 | 20.24 | 12.26 |
| 0104 | 86.05 | 1.11 |
| 0105 | 8.72 | 0.65 |
| 0106 | 175.73 | 6.92 |
| 0107 | 9.6 | 0.64 |
| 0108 | 229.52 | 27.11 |
| 0109 | 92.27 | 6.82 |
| 0110 | 74.49 | 2.11 |
| 0111 | 185.59 | 24.33 |
| 0112 | 105.82 | 5.58 |
| 0113 | 21.6 | 0.13 |
| 0114 | 5.62 | 0.52 |
| 0115 | 93.13 | 3.11 |
| 0116 | 26.33 | 4.86 |
| 0117 | 70.59 | 10.23 |
| 0118 | 42.31 | 1.13 |
| 0119 | 65.73 | 16.51 |
| 0120 | 83.75 | 11.01 |
| 0121 | 184.95 | 1.95 |
| 0122 | 102.92 | 13.52 |
| 0123 | 75.27 | 6.34 |
| 0124 | 240.75 | 18.51 |
| 0125 | 44.24 | 7.81 |
| 0126 | 92.81 | 1.33 |
| 0127 | 139.8 | 1.63 |
| 0128 | 119.26 | 8.31 |
| 0129 | 0 | 0.06 |
| 0130 | 193.43 | 0.83 |
| 0131 | 191.77 | 3.09 |
| 0132 | 101.17 | 5.02 |
| 0133 | 155.32 | 5.27 |
| 0134 | 89.21 | 4.91 |
| 0135 | 82.68 | 14.33 |
| 0136 | 49.88 | 0.5 |
| 0137 | 75.37 | 4.76 |
| 0138 | 9.32 | 11.51 |
| 0139 | 111.85 | 5.53 |
| 0140 | 96.07 | 5.7 |
| 0141 | 54.45 | 2.63 |
| 0142 | 36 | 8.05 |
| 0143 | 73.2 | 27.6 |
| 0144 | 22.03 | 1.45 |
| 0145 | 76.55 | 0.49 |
| 0146 | 41.24 | 0.03 |
| 0147 | 0 | 0.07 |
| 0148 | 70.28 | 4.23 |
| 0149 | 10.26 | 0.82 |
| 0150 | 0 | 0.25 |
| 0151 | 11.12 | 2.08 |
| 0152 | 115.64 | 0.04 |
| 0153 | 0 | 6.28 |
| 0154 | 13.56 | 15.68 |
| 0155 | 0 | 2.03 |
| 0156 | 16.35 | 0.67 |
| 0157 | 298.26 | 8.39 |
| 0158 | 89.28 | 2.75 |
| 0159 | 26.76 | 4.3 |
| 0160 | 55.66 | 27.43 |
| 0161 | 146.77 | 2.05 |
| 0162 | 5.93 | 1.74 |
| 0163 | 0 | 0.61 |
| 0164 | 0 | 9.92 |
| 0165 | 76.52 | 5.29 |
| 0166 | 32.23 | 8.24 |
| 0167 | 10.94 | 2.81 |
| 0168 | 4.49 | 23.75 |
| 0169 | 77.72 | 9.24 |
| 0170 | 39.29 | 9.9 |
| 0171 | 113.4 | 6.92 |
| 0172 | 0 | 13.65 |
| 0173 | 117.72 | 3.56 |
| 0174 | 217.11 | 4.53 |
| 0175 | 195.33 | 0.65 |
| 0176 | 60.77 | 2.1 |
| 0177 | 84.75 | 11.27 |
| 0178 | 164.71 | 23.99 |
| 0179 | 84.44 | 1.24 |
| 0180 | 19.42 | 6.95 |
| 0181 | 176.22 | 3.25 |
| 0182 | 17.74 | 2.84 |
| 0183 | 7.38 | 2.9 |
| 0184 | 104.35 | 10.87 |
| 0185 | 92.19 | 9.24 |
| 0186 | 41.45 | 1.29 |
| 0187 | 30.34 | 4.23 |
| 0188 | 11.13 | 0.03 |
| 0189 | 36.44 | 4.87 |
| 0190 | 52.14 | 6.04 |
| 0191 | 74.49 | 1.36 |
| 0192 | 24.81 | 8.43 |
| 0193 | 50.89 | 30.01 |
| 0194 | 23.53 | 12.29 |
| 0195 | 15.42 | 21.92 |
| 0196 | 7.44 | 17.46 |
| 0197 | 29.68 | 3.95 |
| 0198 | 72.23 | 0.41 |
| 0199 | 60.18 | 9.32 |
| 0200 | 7.44 | 0.41 |
| 0201 | 0 | 0.17 |
| 0202 | 83.58 | 13.81 |
| 0203 | 8.13 | 6.67 |
| 0204 | 139.5 | 25.19 |
| 0205 | 12.87 | 5.04 |
| 0206 | 121.82 | 3 |
| 0207 | 129.88 | 2.72 |
| 0208 | 95.61 | 0.18 |
| 0209 | 15.52 | 0.08 |
| 0210 | 0 | 0.31 |
| 0211 | 8.13 | 0.32 |
| 0212 | 121.28 | 3.96 |
| 0213 | 107.66 | 0.05 |
| 0214 | 80.99 | 4.73 |
| 0215 | 83.53 | 22.83 |
| 0216 | 0 | 0.08 |
| 0217 | 18.87 | 0.03 |
| 0218 | 88.03 | 7.35 |
| 0219 | 68.71 | 5.8 |
| 0220 | 210.1 | 28.25 |
| 0221 | 98.57 | 0.43 |
| 0222 | 227.07 | 14.58 |
| 0223 | 155.73 | 4.8 |
| 0224 | 17.65 | 1.64 |
| 0225 | 154.09 | 15.11 |
| 0226 | 41.37 | 1.47 |
| 0227 | 114.48 | 5.67 |
| 0228 | 28.79 | 0.81 |
| 0229 | 23.93 | 0.98 |
| 0230 | 16.1 | 0.26 |
| 0231 | 45.65 | 25.23 |
| 0232 | 81.11 | 2.76 |
| 0233 | 0 | 2.58 |
| 0234 | 60.73 | 2.63 |
| 0235 | 104.69 | 0.26 |
| 0236 | 55.58 | 7.54 |
| 0237 | 9.5 | 0.07 |
| 0238 | 50.4 | 6.81 |
| 0239 | 72.54 | 18.58 |
| 0240 | 58.85 | 3.28 |
| 0241 | 16.74 | 0.31 |
| 0242 | 194.23 | 9 |
| 0243 | 108.16 | 26.41 |
| 0244 | 20.65 | 4.48 |
| 0245 | 42.04 | 24.85 |
| 0246 | 8.78 | 1.56 |
| 0247 | 30.21 | 1.88 |
| 0248 | 119.36 | 15.12 |
| 0249 | 146.22 | 7.91 |
| 0250 | 68.39 | 5.13 |
| 0251 | 44.29 | 2.41 |
| 0252 | 5.78 | 3.05 |
| 0253 | 100.66 | 21.64 |
| 0254 | 226.86 | 28.98 |
| 0255 | 32.63 | 3.01 |
| 0256 | 64.81 | 3.48 |
| 0257 | 192.45 | 0.87 |
| 0258 | 0 | 20.67 |
| 0259 | 35.65 | 5.71 |
| 0260 | 67.83 | 10.8 |
| 0261 | 59.97 | 5.4 |
| 0262 | 0 | 0.15 |
| 0263 | 17.2 | 16.39 |
| 0264 | 0 | 0.62 |
| 0265 | 250.49 | 7.31 |
| 0266 | 235.36 | 5.39 |
| 0267 | 109.09 | 1.59 |
| 0268 | 18.14 | 0.76 |
| 0269 | 8.4 | 6.83 |
| 0270 | 22.08 | 12.25 |
| 0271 | 113.54 | 1.83 |
| 0272 | 88.57 | 3.02 |
| 0273 | 33.42 | 2.43 |
| 0274 | 116.13 | 9.22 |
| 0275 | 36.38 | 1.22 |
| 0276 | 259.87 | 22.34 |
| 0277 | 10.71 | 1.52 |
| 0278 | 95.97 | 7.84 |
| 0279 | 16.08 | 0.55 |
| 0280 | 48.35 | 0.18 |
| 0281 | 117.08 | 1.33 |
| 0282 | 56.17 | 1.77 |
| 0283 | 61.71 | 1.51 |
| 0284 | 5.94 | 40.46 |
| 0285 | 148.16 | 1.52 |
| 0286 | 41.37 | 0.35 |
| 0287 | 109.09 | 19.79 |
| 0288 | 14.3 | 1.25 |
| 0289 | 196.51 | 20.04 |
| 0290 | 85.23 | 12.85 |
| 0291 | 19.3 | 16.44 |
| 0292 | 147.57 | 6.96 |
| 0293 | 112.19 | 2.45 |
| 0294 | 18 | 1.53 |
| 0295 | 70.38 | 0.19 |
| 0296 | 61.62 | 0.06 |
| 0297 | 72.16 | 2.19 |
| 0298 | 33.12 | 0.45 |
| 0299 | 99.4 | 9.76 |
| 0300 | 13.91 | 15.31 |
| 0301 | 13.6 | 3.76 |
| 0302 | 34.8 | 12.27 |
| 0303 | 0 | 3.82 |
| 0304 | 26.95 | 0.87 |
| 0305 | 7.56 | 25.45 |
| 0306 | 0 | 4.06 |
| 0307 | 39.58 | 5.54 |
| 0308 | 84.61 | 5.51 |
| 0309 | 75.31 | 2.51 |
| 0310 | 87.12 | 14.9 |
| 0311 | 83.55 | 16.15 |
| 0312 | 8.06 | 6.49 |
| 0313 | 61.02 | 0 |
| 0314 | 95.5 | 0.15 |
| 0315 | 241.08 | 20.52 |
| 0316 | 72.2 | 9.29 |
| 0317 | 105.87 | 36.56 |
| 0318 | 160.39 | 0.07 |
| 0319 | 87.85 | 25.97 |
| 0320 | 98.72 | 2.65 |
| 0321 | 61.49 | 0.33 |
| 0322 | 106.49 | 5.04 |
| 0323 | 217.06 | 6.48 |
| 0324 | 73.34 | 23.2 |
| 0325 | 34.09 | 6.77 |
| 0326 | 83.05 | 1.36 |
| 0327 | 8.12 | 0.94 |
| 0328 | 124.93 | 9.89 |
| 0329 | 37.17 | 6.18 |
| 0330 | 92.3 | 1.55 |
| 0331 | 253.77 | 3.07 |
| 0332 | 93.88 | 8.8 |
| 0333 | 148.39 | 35.5 |
| 0334 | 114.92 | 23.26 |
| 0335 | 73.87 | 2.76 |
| 0336 | 80.28 | 0.24 |
| 0337 | 15.78 | 2.86 |
| 0338 | 95.05 | 1.6 |
| 0339 | 163.66 | 4.61 |
| 0340 | 104.54 | 3.39 |
| 0341 | 93.48 | 2.4 |
| 0342 | 49.97 | 1.73 |
| 0343 | 111.33 | 23.57 |
| 0344 | 83.47 | 0.59 |
| 0345 | 99.98 | 9.71 |
| 0346 | 64.75 | 5.67 |
| 0347 | 81.03 | 6.07 |
| 0348 | 14.61 | 10.6 |
| 0349 | 0 | 0.06 |
| 0350 | 16.82 | 1.5 |
| 0351 | 41.03 | 31.63 |
| 0352 | 10.86 | 0.04 |
| 0353 | 78.66 | 10.27 |
| 0354 | 5.2 | 1.81 |
| 0355 | 55.29 | 18.07 |
| 0356 | 129.88 | 9.94 |
| 0357 | 162.58 | 7.82 |
| 0358 | 81.27 | 2.68 |
| 0359 | 0 | 0.99 |
| 0360 | 0 | 15 |
| 0361 | 42.17 | 1.67 |
| 0362 | 20.97 | 7.59 |
| 0363 | 5.58 | 0.15 |
| 0364 | 0 | 0.3 |
| 0365 | 137.45 | 6.34 |
| 0366 | 52.07 | 1.41 |
| 0367 | 149.2 | 38.2 |
| 0368 | 0 | 3.86 |
| 0369 | 11.26 | 5.4 |
| 0370 | 73.42 | 0.05 |
| 0371 | 88.15 | 0.21 |
| 0372 | 20.95 | 0.58 |
| 0373 | 46.44 | 6.18 |
| 0374 | 76.64 | 4.45 |
| 0375 | 67.21 | 1.74 |
| 0376 | 97.33 | 1.5 |
| 0377 | 64.46 | 2.81 |
| 0378 | 46.29 | 4.89 |
| 0379 | 0 | 13.86 |
| 0380 | 46.82 | 1.55 |
| 0381 | 218.96 | 12.63 |
| 0382 | 15.14 | 0.23 |
| 0383 | 24.18 | 0.49 |
| 0384 | 76.68 | 19.22 |
| 0385 | 87.15 | 27.04 |
| 0386 | 60.97 | 1.82 |
| 0387 | 57.58 | 29.55 |
| 0388 | 7.24 | 10.79 |
| 0389 | 83.99 | 12.6 |
| 0390 | 0 | 13.97 |
| 0391 | 0 | 24.37 |
| 0392 | 152.39 | 20.75 |
| 0393 | 29.67 | 0.16 |
| 0394 | 7.47 | 25.89 |
| 0395 | 48.89 | 8.88 |
| 0396 | 5.72 | 8.32 |
| 0397 | 72.87 | 25.05 |
| 0398 | 15.67 | 4.53 |
| 0399 | 101.04 | 10.65 |
| 0400 | 373.09 | 8.62 |
| 0401 | 5.47 | 4.61 |
| 0402 | 0 | 0.14 |
| 0403 | 7.19 | 0.06 |
| 0404 | 52.58 | 2.26 |
| 0405 | 222.07 | 9.68 |
| 0406 | 95.46 | 5.77 |
| 0407 | 15.05 | 7.57 |
| 0408 | 15.51 | 1.68 |
| 0409 | 25.42 | 0.01 |
| 0410 | 44.97 | 10.61 |
| 0411 | 177.38 | 15.93 |
| 0412 | 55.37 | 13.17 |
| 0413 | 48.32 | 14.34 |
| 0414 | 90.26 | 3.05 |
| 0415 | 63.7 | 5.13 |
| 0416 | 108.98 | 32.42 |
| 0417 | 113.83 | 14.26 |
| 0418 | 31.29 | 10.13 |
| 0419 | 86.5 | 2 |
| 0420 | 10.17 | 7.89 |
| 0421 | 41.63 | 0.8 |
| 0422 | 62.39 | 0.19 |
| 0423 | 23.88 | 0.51 |
| 0424 | 46.92 | 6.1 |
| 0425 | 137.37 | 5.71 |
| 0426 | 11.84 | 0.31 |
| 0427 | 87.83 | 9.29 |
| 0428 | 39.96 | 3.72 |
| 0429 | 6.04 | 3.1 |
| 0430 | 9.46 | 0.3 |
| 0431 | 36.34 | 10.85 |
| 0432 | 129.24 | 26.02 |
| 0433 | 33.36 | 5.21 |
| 0434 | 90.22 | 4.81 |
| 0435 | 16.59 | 8.79 |
| 0436 | 114.84 | 11.31 |
| 0437 | 0 | 0.04 |
| 0438 | 65.34 | 14.09 |
| 0439 | 10.81 | 10.84 |
| 0440 | 74.68 | 9.01 |
| 0441 | 7.37 | 1.32 |
| 0442 | 10.13 | 0.02 |
| 0443 | 125.59 | 0.1 |
| 0444 | 69.45 | 41.92 |
| 0445 | 142.09 | 5.77 |
| 0446 | 7.88 | 26.97 |
| 0447 | 20.23 | 3.18 |
| 0448 | 74.67 | 0.92 |
| 0449 | 84.16 | 4.52 |
| 0450 | 52.65 | 7.56 |
| 0451 | 98.62 | 12.04 |
| 0452 | 39.87 | 0.74 |
| 0453 | 107.08 | 17.9 |
| 0454 | 28.42 | 0.03 |
| 0455 | 117.34 | 9.65 |
| 0456 | 7.48 | 3.79 |
| 0457 | 96.38 | 21.88 |
| 0458 | 0 | 11.23 |
| 0459 | 123.82 | 4.33 |
| 0460 | 45.22 | 3.55 |
| 0461 | 99.02 | 12.1 |
| 0462 | 32.89 | 1.11 |
| 0463 | 43.32 | 0.98 |
| 0464 | 180.54 | 11.38 |
| 0465 | 73.71 | 3.71 |
| 0466 | 132.61 | 6.17 |
| 0467 | 163.56 | 9.32 |
| 0468 | 132.41 | 3.45 |
| 0469 | 5.86 | 0.41 |
| 0470 | 24.93 | 0.33 |
| 0471 | 95.36 | 17.03 |
| 0472 | 19.96 | 9.61 |
| 0473 | 0 | 0.9 |
| 0474 | 52.21 | 1.26 |
| 0475 | 8.13 | 14.32 |
| 0476 | 65 | 19.17 |
| 0477 | 59.04 | 1.23 |
| 0478 | 6.01 | 8.38 |
| 0479 | 38.63 | 3.97 |
| 0480 | 121.97 | 3.19 |
| 0481 | 15.67 | 6.07 |
| 0482 | 41.09 | 0.18 |
| 0483 | 139.87 | 1.44 |
| 0484 | 0 | 6.53 |
| 0485 | 258.4 | 0.31 |
| 0486 | 120.15 | 9.21 |
| 0487 | 101.92 | 11.01 |
| 0488 | 91.83 | 0.04 |
| 0489 | 7.33 | 10.3 |
| 0490 | 76.27 | 1.01 |
| 0491 | 37.01 | 10.13 |
| 0492 | 90.15 | 0.7 |
| 0493 | 422.72 | 32.3 |
| 0494 | 7.41 | 2.71 |
| 0495 | 49.81 | 1.4 |
| 0496 | 8.42 | 2.69 |
| 0497 | 33.23 | 4.67 |
| 0498 | 108.05 | 5.24 |
| 0499 | 122.76 | 0.32 |
| 0500 | 36.6 | 12.07 |
| 0501 | 17.04 | 0.04 |
| 0502 | 102.05 | 15.32 |
| 0503 | 94.22 | 8.51 |
| 0504 | 62.22 | 0.75 |
| 0505 | 93.77 | 0.04 |
| 0506 | 20.01 | 0.18 |
| 0507 | 0 | 0.03 |
| 0508 | 45.2 | 3.7 |
| 0509 | 0 | 1.68 |
| 0510 | 19.24 | 15.14 |
| 0511 | 92.65 | 6.29 |
| 0512 | 13.88 | 9.67 |
| 0513 | 10.61 | 8.43 |
| 0514 | 66.96 | 0.03 |
| 0515 | 8.19 | 9.33 |
| 0516 | 145.13 | 0.97 |
| 0517 | 228.56 | 1.82 |
| 0518 | 50.69 | 4.88 |
| 0519 | 81.83 | 2.47 |
| 0520 | 65.01 | 0.14 |
| 0521 | 14.49 | 2.62 |
| 0522 | 125.06 | 11.4 |
| 0523 | 106.83 | 6.5 |
| 0524 | 69.77 | 0.88 |
| 0525 | 48.04 | 7.78 |
| 0526 | 34.07 | 21.27 |
| 0527 | 36.9 | 5.75 |
| 0528 | 7.41 | 0.04 |
| 0529 | 167.19 | 17.75 |
| 0530 | 111.48 | 0.07 |
| 0531 | 324.68 | 17.31 |
| 0532 | 24.48 | 2.08 |
| 0533 | 45.9 | 1.86 |
| 0534 | 49.12 | 15.4 |
| 0535 | 37.07 | 14.42 |
| 0536 | 33.04 | 2.11 |
| 0537 | 94.37 | 1.54 |
| 0538 | 27.46 | 5.63 |
| 0539 | 155.68 | 0.06 |
| 0540 | 30.27 | 8.42 |
| 0541 | 67.24 | 38.16 |
| 0542 | 65.02 | 0.65 |
| 0543 | 6.62 | 1.05 |
| 0544 | 45.21 | 4.63 |
| 0545 | 93.76 | 0.04 |
| 0546 | 4.39 | 2.25 |
| 0547 | 8.44 | 6.96 |
| 0548 | 15.24 | 1.54 |
| 0549 | 11.73 | 0.92 |
| 0550 | 32.24 | 1.08 |
| 0551 | 22.84 | 16.56 |
| 0552 | 5.32 | 5.35 |
| 0553 | 23.46 | 3.93 |
| 0554 | 67.66 | 0.25 |
| 0555 | 70.8 | 3.88 |
| 0556 | 86.38 | 3.22 |
| 0557 | 116.56 | 8.1 |
| 0558 | 38.33 | 0.4 |
| 0559 | 170.43 | 4.98 |
| 0560 | 65.04 | 11.13 |
| 0561 | 203.93 | 8.77 |
| 0562 | 0 | 12.29 |
| 0563 | 6.76 | 9.76 |
| 0564 | 17.95 | 10.26 |
| 0565 | 199.91 | 3.01 |
| 0566 | 22.09 | 0.06 |
| 0567 | 72.77 | 2.23 |
| 0568 | 35.15 | 0.42 |
| 0569 | 47.9 | 4.81 |
| 0570 | 37.7 | 11.51 |
| 0571 | 117.49 | 1.08 |
| 0572 | 17.26 | 0.4 |
| 0573 | 83.04 | 4.57 |
| 0574 | 107.29 | 3.92 |
| 0575 | 137.41 | 4.26 |
| 0576 | 0 | 4.42 |
| 0577 | 0 | 4.81 |
| 0578 | 0 | 0.06 |
| 0579 | 144.25 | 3.79 |
| 0580 | 45.15 | 10.55 |
| 0581 | 103.19 | 1.39 |
| 0582 | 0 | 5.23 |
| 0583 | 30.08 | 12.74 |
| 0584 | 66.96 | 0.59 |
| 0585 | 131.42 | 0.22 |
| 0586 | 20.35 | 4.07 |
| 0587 | 55.89 | 1.94 |
| 0588 | 0 | 4.28 |
| 0589 | 70.23 | 2.59 |
| 0590 | 0 | 1.46 |
| 0591 | 215.08 | 12.99 |
| 0592 | 13.64 | 2.9 |
| 0593 | 35.57 | 1.43 |
| 0594 | 30.85 | 2.91 |
| 0595 | 0 | 20.12 |
| 0596 | 100.98 | 0.07 |
| 0597 | 182.11 | 9.13 |
| 0598 | 92.03 | 2.69 |
| 0599 | 17.3 | 12.63 |
| 0600 | 76.73 | 2 |
| 0601 | 79.09 | 15.55 |
| 0602 | 8.7 | 11.6 |
| 0603 | 113.96 | 1.04 |
| 0604 | 148.37 | 2.66 |
| 0605 | 76.5 | 0.54 |
| 0606 | 0 | 18.15 |
| 0607 | 124.86 | 5.94 |
| 0608 | 111.05 | 9.79 |
| 0609 | 3.85 | 7.52 |
| 0610 | 114.06 | 7.2 |
| 0611 | 13.62 | 3.34 |
| 0612 | 61.92 | 9.85 |
| 0613 | 9.36 | 6.94 |
| 0614 | 0 | 2.23 |
| 0615 | 31.32 | 5.3 |
| 0616 | 20.55 | 4.17 |
| 0617 | 76.9 | 4.8 |
| 0618 | 0 | 1.8 |
| 0619 | 36.4 | 0.11 |
| 0620 | 112.95 | 1.03 |
| 0621 | 82.85 | 3.18 |
| 0622 | 17.16 | 0.76 |
| 0623 | 201.33 | 2.56 |
| 0624 | 25.95 | 1.4 |
| 0625 | 18.79 | 26.89 |
| 0626 | 15.11 | 11.11 |
| 0627 | 18.18 | 10.64 |
| 0628 | 49.12 | 1.54 |
| 0629 | 35.69 | 7.2 |
| 0630 | 44.83 | 6.5 |
| 0631 | 6.06 | 8.68 |
| 0632 | 271.03 | 5.97 |
| 0633 | 160.67 | 6.45 |
| 0634 | 65.76 | 0.25 |
| 0635 | 168.18 | 8.56 |
| 0636 | 38.34 | 3.39 |
| 0637 | 39.06 | 2.37 |
| 0638 | 13.29 | 6.17 |
| 0639 | 68.33 | 0.02 |
| 0640 | 77.82 | 0.11 |
| 0641 | 222.45 | 15.48 |
| 0642 | 0 | 0.1 |
| 0643 | 11.97 | 0.71 |
| 0644 | 7.03 | 7.14 |
| 0645 | 5.73 | 0.08 |
| 0646 | 13.46 | 20.39 |
| 0647 | 86.64 | 6.16 |
| 0648 | 128.65 | 5.11 |
| 0649 | 123.43 | 12.96 |
| 0650 | 0 | 7.46 |
| 0651 | 39.47 | 0.69 |
| 0652 | 47.16 | 0.07 |
| 0653 | 12.25 | 6.75 |
| 0654 | 72.45 | 2.87 |
| 0655 | 38.07 | 17.01 |
| 0656 | 120.61 | 2.09 |
| 0657 | 11.35 | 4.7 |
| 0658 | 9.15 | 2.22 |
| 0659 | 17.57 | 0.93 |
| 0660 | 0 | 0.27 |
| 0661 | 68.02 | 3.23 |
| 0662 | 107.4 | 21.95 |
| 0663 | 123.23 | 3.1 |
| 0664 | 0 | 1.98 |
| 0665 | 0 | 0.05 |
| 0666 | 161.35 | 31.67 |
| 0667 | 111.55 | 9.37 |
| 0668 | 75.82 | 4.73 |
| 0669 | 13.65 | 10.21 |
| 0670 | 101.16 | 2.42 |
| 0671 | 113.55 | 1.15 |
| 0672 | 21.79 | 1.69 |
| 0673 | 65.41 | 11.71 |
| 0674 | 7.92 | 3.41 |
| 0675 | 124.91 | 0.65 |
| 0676 | 25.5 | 0.06 |
| 0677 | 5.99 | 3.85 |
| 0678 | 144.3 | 0.08 |
| 0679 | 35.61 | 1.5 |
| 0680 | 87.66 | 0.06 |
| 0681 | 163.77 | 4.12 |
| 0682 | 37.4 | 11.78 |
| 0683 | 68.82 | 0.25 |
| 0684 | 45.31 | 8.01 |
| 0685 | 11.81 | 2.54 |
| 0686 | 71.89 | 0.58 |
| 0687 | 169.23 | 5.41 |
| 0688 | 11.86 | 8.21 |
| 0689 | 66.95 | 4.2 |
| 0690 | 13.6 | 4.74 |
| 0691 | 67.18 | 4.18 |
| 0692 | 91.05 | 10.11 |
| 0693 | 93.95 | 0.07 |
| 0694 | 4.64 | 0.1 |
| 0695 | 178.94 | 36.73 |
| 0696 | 122.74 | 3.62 |
| 0697 | 82.18 | 4.91 |
| 0698 | 7.99 | 2.12 |
| 0699 | 7.36 | 28.07 |
| 0700 | 29 | 0.63 |
| 0701 | 6.12 | 5.06 |
| 0702 | 65.35 | 9.75 |
| 0703 | 66.33 | 0.02 |
| 0704 | 32.17 | 0.4 |
| 0705 | 23.48 | 4.23 |
| 0706 | 62.66 | 9.11 |
| 0707 | 29.37 | 0.24 |
| 0708 | 101.68 | 0.96 |
| 0709 | 31.7 | 4.54 |
| 0710 | 275.83 | 11.88 |
| 0711 | 41 | 21.05 |
| 0712 | 34.88 | 1.03 |
| 0713 | 6.29 | 0.72 |
| 0714 | 0 | 7.66 |
| 0715 | 106.17 | 0.51 |
| 0716 | 81.71 | 5.19 |
| 0717 | 203.17 | 4.27 |
| 0718 | 62.97 | 0.47 |
| 0719 | 147.73 | 17.37 |
| 0720 | 0 | 0.49 |
| 0721 | 3.67 | 3.44 |
| 0722 | 16.89 | 0.48 |
| 0723 | 12.94 | 10.79 |
| 0724 | 0 | 4.68 |
| 0725 | 75.48 | 0.27 |
| 0726 | 133.88 | 24.54 |
| 0727 | 79.59 | 8.57 |
| 0728 | 94.96 | 6.4 |
| 0729 | 62.6 | 1.09 |
| 0730 | 200.84 | 5.84 |
| 0731 | 207.57 | 0.83 |
| 0732 | 15.08 | 5.76 |
| 0733 | 148.86 | 9.98 |
| 0734 | 0 | 0.49 |
| 0735 | 20.12 | 0.67 |
| 0736 | 60.42 | 11.41 |
| 0737 | 70.04 | 17.12 |
| 0738 | 163.52 | 3.2 |
| 0739 | 15.61 | 1.27 |
| 0740 | 21.13 | 13.08 |
| 0741 | 22.87 | 1.29 |
| 0742 | 34.18 | 0.02 |
| 0743 | 156.9 | 0.12 |
| 0744 | 185.95 | 4.83 |
| 0745 | 94.15 | 7.17 |
| 0746 | 83.47 | 5.84 |
| 0747 | 0 | 22.63 |
| 0748 | 63.92 | 5.08 |
| 0749 | 7.57 | 1.2 |
| 0750 | 9.51 | 3.7 |
| 0751 | 67.71 | 2.79 |
| 0752 | 122.12 | 5.88 |
| 0753 | 31.26 | 1.05 |
| 0754 | 112.89 | 2.22 |
| 0755 | 141.49 | 0.37 |
| 0756 | 76.73 | 1.31 |
| 0757 | 6.21 | 0.07 |
| 0758 | 0 | 2.71 |
| 0759 | 135.51 | 5.7 |
| 0760 | 59.58 | 6.37 |
| 0761 | 0 | 5.36 |
| 0762 | 0 | 1.03 |
| 0763 | 7.45 | 27.76 |
| 0764 | 0 | 0.83 |
| 0765 | 70.53 | 7.39 |
| 0766 | 0 | 3.74 |
| 0767 | 60.19 | 1.92 |
| 0768 | 204.95 | 7.27 |
| 0769 | 88.7 | 0.07 |
| 0770 | 99.7 | 16.56 |
| 0771 | 6.85 | 6.27 |
| 0772 | 59.82 | 1.27 |
| 0773 | 0 | 14.81 |
| 0774 | 55.79 | 9.99 |
| 0775 | 48.57 | 10.18 |
| 0776 | 17.65 | 6.5 |
| 0777 | 28.01 | 6.1 |
| 0778 | 68.33 | 4.08 |
| 0779 | 73.53 | 0.06 |
| 0780 | 49.93 | 1.43 |
| 0781 | 10.27 | 0.11 |
| 0782 | 71.29 | 2.82 |
| 0783 | 13.15 | 6.35 |
| 0784 | 86.89 | 8.42 |
| 0785 | 7.25 | 1.66 |
| 0786 | 140.91 | 2.83 |
| 0787 | 70.46 | 6.11 |
| 0788 | 0 | 0.08 |
| 0789 | 7.26 | 1.82 |
| 0790 | 12.97 | 0.07 |
| 0791 | 26.87 | 7.4 |
| 0792 | 195.25 | 2.8 |
| 0793 | 103.66 | 0.89 |
| 0794 | 245.6 | 0.52 |
| 0795 | 0 | 12.39 |
| 0796 | 50.09 | 10.25 |
| 0797 | 77.23 | 17.5 |
| 0798 | 0 | 4.6 |
| 0799 | 0 | 0.11 |
| 0800 | 202.26 | 0.33 |
| 0801 | 0 | 6.13 |
| 0802 | 27.28 | 2.16 |
| 0803 | 99.49 | 7.12 |
| 0804 | 28.81 | 1.49 |
| 0805 | 65.52 | 4.55 |
| 0806 | 90.78 | 12.53 |
| 0807 | 94.71 | 5.79 |
| 0808 | 121.99 | 11.25 |
| 0809 | 58.6 | 15.1 |
| 0810 | 63.51 | 3.72 |
| 0811 | 31.06 | 0.31 |
| 0812 | 42.59 | 12.02 |
| 0813 | 78.93 | 17.2 |
| 0814 | 246.44 | 30.72 |
| 0815 | 46.39 | 9.96 |
| 0816 | 67.9 | 0.18 |
| 0817 | 37.63 | 3.65 |
| 0818 | 28.57 | 0.12 |
| 0819 | 68.21 | 27.01 |
| 0820 | 92.68 | 2.07 |
| 0821 | 10 | 15.19 |
| 0822 | 149.22 | 0.6 |
| 0823 | 179.12 | 4.6 |
| 0824 | 42.33 | 4.44 |
| 0825 | 335.28 | 5.24 |
| 0826 | 65.63 | 6.11 |
| 0827 | 105.18 | 3.22 |
| 0828 | 6.88 | 5.31 |
| 0829 | 53.95 | 8.61 |
| 0830 | 28.89 | 8.43 |
| 0831 | 0 | 3.55 |
| 0832 | 157.32 | 2.47 |
| 0833 | 20.07 | 10.44 |
| 0834 | 138.2 | 10.6 |
| 0835 | 171.63 | 18.39 |
| 0836 | 92.03 | 4.12 |
| 0837 | 6 | 3.42 |
| 0838 | 5.01 | 6.89 |
| 0839 | 31.64 | 5.53 |
| 0840 | 17.83 | 6.89 |
| 0841 | 114.34 | 0.48 |
| 0842 | 95.43 | 0.29 |
| 0843 | 0 | 0.09 |
| 0844 | 14.37 | 0.1 |
| 0845 | 111.94 | 17.21 |
| 0846 | 39.18 | 9.14 |
| 0847 | 43.46 | 0.52 |
| 0848 | 96.51 | 37.29 |
| 0849 | 32.99 | 20.32 |
| 0850 | 94.56 | 4.62 |
| 0851 | 15.37 | 4.43 |
| 0852 | 45.85 | 16.57 |
| 0853 | 109.34 | 1.65 |
| 0854 | 91.35 | 20.73 |
| 0855 | 42.11 | 3.68 |
| 0856 | 14.17 | 2.26 |
| 0857 | 44.92 | 2.85 |
| 0858 | 30.47 | 8.42 |
| 0859 | 117.24 | 11.34 |
| 0860 | 35.52 | 1.12 |
| 0861 | 106.6 | 3.61 |
| 0862 | 26.46 | 6.5 |
| 0863 | 50.12 | 13.83 |
| 0864 | 106.9 | 1.43 |
| 0865 | 201.31 | 3.53 |
| 0866 | 75.33 | 6.99 |
| 0867 | 50.26 | 0.07 |
| 0868 | 0 | 1.81 |
| 0869 | 77.61 | 0.16 |
| 0870 | 76.27 | 5.22 |
| 0871 | 93.4 | 2.76 |
| 0872 | 86.22 | 3.21 |
| 0873 | 39.77 | 0.57 |
| 0874 | 128.22 | 1.45 |
| 0875 | 40.49 | 10.4 |
| 0876 | 17.09 | 23.43 |
| 0877 | 131.34 | 0.71 |
| 0878 | 47.22 | 2.93 |
| 0879 | 0 | 2.63 |
| 0880 | 82.73 | 6.72 |
| 0881 | 0 | 0.06 |
| 0882 | 116.27 | 16.47 |
| 0883 | 0 | 0.06 |
| 0884 | 88.49 | 5.13 |
| 0885 | 14.98 | 0.08 |
| 0886 | 60.21 | 9.7 |
| 0887 | 61 | 3.29 |
| 0888 | 166.72 | 16.95 |
| 0889 | 65.49 | 0.75 |
| 0890 | 217.15 | 7.48 |
| 0891 | 105.4 | 5.58 |
| 0892 | 0 | 0.97 |
| 0893 | 91.64 | 37.91 |
| 0894 | 104.93 | 0.48 |
| 0895 | 7.41 | 0.02 |
| 0896 | 88.01 | 4.59 |
| 0897 | 50.95 | 12.85 |
| 0898 | 0 | 0.14 |
| 0899 | 0 | 5.83 |
| 0900 | 105.03 | 11.37 |
| 0901 | 148.35 | 0.68 |
| 0902 | 43.45 | 4.9 |
| 0903 | 0 | 3.37 |
| 0904 | 7.1 | 7.52 |
| 0905 | 157.5 | 18.28 |
| 0906 | 6.98 | 6.96 |
| 0907 | 71.94 | 17.01 |
| 0908 | 175.56 | 1.54 |
| 0909 | 99.12 | 42.78 |
| 0910 | 123.01 | 1.64 |
| 0911 | 13.26 | 0.12 |
| 0912 | 97.83 | 35.54 |
| 0913 | 73.86 | 5.83 |
| 0914 | 127.97 | 7.39 |
| 0915 | 14.79 | 8.59 |
| 0916 | 141.11 | 3.34 |
| 0917 | 143.13 | 2.31 |
| 0918 | 0 | 0.16 |
| 0919 | 85.55 | 2.14 |
| 0920 | 5.9 | 1.51 |
| 0921 | 38.02 | 19.27 |
| 0922 | 83.67 | 9.67 |
| 0923 | 146.48 | 0.53 |
| 0924 | 55.12 | 0.01 |
| 0925 | 107.48 | 0.6 |
| 0926 | 36.23 | 1.21 |
| 0927 | 87.51 | 1.25 |
| 0928 | 20.64 | 0.28 |
| 0929 | 27.87 | 0.02 |
| 0930 | 144.98 | 0.68 |
| 0931 | 98.91 | 7.26 |
| 0932 | 88.76 | 18.6 |
| 0933 | 43.09 | 1.57 |
| 0934 | 25.83 | 7.72 |
| 0935 | 12.46 | 3.64 |
| 0936 | 79.75 | 5.96 |
| 0937 | 27.03 | 11.31 |
| 0938 | 53.07 | 0.02 |
| 0939 | 12.73 | 0.32 |
| 0940 | 175.47 | 7.55 |
| 0941 | 71.94 | 3.68 |
| 0942 | 28.22 | 15.21 |
| 0943 | 63.91 | 0.66 |
| 0944 | 0 | 11.41 |
| 0945 | 0 | 13.4 |
| 0946 | 89.49 | 10.88 |
| 0947 | 10.28 | 5.89 |
| 0948 | 161.84 | 5.24 |
| 0949 | 27.92 | 0.17 |
| 0950 | 129.12 | 40.93 |
| 0951 | 35.68 | 0.28 |
| 0952 | 163.82 | 12.97 |
| 0953 | 7.77 | 6.77 |
| 0954 | 46.99 | 1.73 |
| 0955 | 0 | 0.1 |
| 0956 | 93.16 | 2.57 |
| 0957 | 55.86 | 4.38 |
| 0958 | 40.14 | 29.71 |
| 0959 | 88.75 | 8.89 |
| 0960 | 5.97 | 2.82 |
| 0961 | 59.05 | 4.27 |
| 0962 | 36.66 | 5.89 |
| 0963 | 103.62 | 3.11 |
| 0964 | 129.94 | 12.31 |
| 0965 | 130.03 | 4.5 |
| 0966 | 51.64 | 3.81 |
| 0967 | 74.01 | 9.45 |
| 0968 | 103.09 | 6.12 |
| 0969 | 87.4 | 17.78 |
| 0970 | 34.64 | 3.25 |
| 0971 | 85.54 | 0.12 |
| 0972 | 11.28 | 1.54 |
| 0973 | 140.64 | 2.47 |
| 0974 | 0 | 21.56 |
| 0975 | 39.89 | 0.82 |
| 0976 | 24.17 | 5.46 |
| 0977 | 75.52 | 1.93 |
| 0978 | 21.41 | 6.16 |
| 0979 | 103.18 | 8.62 |
| 0980 | 78.86 | 3.85 |
| 0981 | 104.69 | 21.59 |
| 0982 | 126.08 | 17.82 |
| 0983 | 52.62 | 4.21 |
| 0984 | 5.74 | 0.93 |
| 0985 | 0 | 3.92 |
| 0986 | 0 | 10.78 |
| 0987 | 15.05 | 6.87 |
| 0988 | 150.21 | 0.22 |
| 0989 | 113.49 | 7.94 |
| 0990 | 82.3 | 3.96 |
| 0991 | 29.6 | 0.1 |
| 0992 | 68.99 | 0.13 |
| 0993 | 30.05 | 2.46 |
| 0994 | 127.98 | 1.57 |
| 0995 | 74.66 | 4 |
| 0996 | 12.63 | 2.18 |
| 0997 | 75.67 | 4.27 |
| 0998 | 36.29 | 0.08 |
| 0999 | 11.8 | 14.58 |
| 1000 | 170.49 | 12.05 |
| 1001 | 102.54 | 2.4 |
| 1002 | 11.96 | 0.1 |
| 1003 | 0 | 4.4 |
| 1004 | 103.99 | 2.13 |
| 1005 | 67.82 | 19.76 |
| 1006 | 241.57 | 0.11 |
| 1007 | 0 | 0.07 |
| 1008 | 14.54 | 0.07 |
| 1009 | 97.71 | 4.77 |
| 1010 | 123.67 | 28.48 |
| 1011 | 0 | 2.4 |
| 1012 | 46.01 | 1.52 |
| 1013 | 106.7 | 1.93 |
| 1014 | 10.24 | 1.27 |
| 1015 | 146.9 | 1.66 |
| 1016 | 84.8 | 0.57 |
| 1017 | 0 | 0.33 |
| 1018 | 12.59 | 0.72 |
| 1019 | 69.19 | 0.9 |
| 1020 | 83.71 | 4 |
| 1021 | 107.44 | 0.84 |
| 1022 | 8.1 | 1.87 |
| 1023 | 120.22 | 19.46 |
| 1024 | 69.2 | 10.15 |
| 1025 | 45.18 | 15.21 |
| 1026 | 143.94 | 3.38 |
| 1027 | 23.25 | 7.97 |
| 1028 | 328.83 | 6.52 |
| 1029 | 81.78 | 5.39 |
| 1030 | 0 | 15.71 |
| 1031 | 0 | 0.65 |
| 1032 | 63.46 | 13.72 |
| 1033 | 87.81 | 32.02 |
| 1034 | 67.73 | 7.99 |
| 1035 | 86.67 | 2.88 |
| 1036 | 80.1 | 0.31 |
| 1037 | 0 | 7.55 |
| 1038 | 51.41 | 12.12 |
| 1039 | 27.01 | 21.83 |
| 1040 | 82.96 | 2.3 |
| 1041 | 9.13 | 0.34 |
| 1042 | 18.95 | 14.58 |
| 1043 | 112.52 | 4.11 |
| 1044 | 48.7 | 6.36 |
| 1045 | 66.57 | 4.06 |
| 1046 | 56.67 | 1.41 |
| 1047 | 6.35 | 9.52 |
| 1048 | 86.48 | 0.73 |
| 1049 | 30.11 | 0.18 |
| 1050 | 7.16 | 0.34 |
| 1051 | 18.61 | 0.48 |
| 1052 | 72.7 | 2.04 |
| 1053 | 73.89 | 2.68 |
| 1054 | 38.32 | 2.47 |
| 1055 | 0 | 3.96 |
| 1056 | 39.07 | 4.46 |
| 1057 | 101.5 | 13.36 |
| 1058 | 12.44 | 0.89 |
| 1059 | 6.55 | 0.54 |
| 1060 | 10.68 | 1.62 |
| 1061 | 70.55 | 5.97 |
| 1062 | 155.24 | 3.64 |
| 1063 | 83.86 | 24.55 |
| 1064 | 135.3 | 5.1 |
| 1065 | 10.82 | 0.22 |
| 1066 | 107.37 | 0.64 |
| 1067 | 190.65 | 13.58 |
| 1068 | 66.1 | 0.84 |
